# Minimum 5-Year Follow-Up Assessment of Volar Plate Interposition Arthroplasty for Post-Traumatic Osteoarthritis in Proximal Interphalangeal Joints

**DOI:** 10.3390/jcm12144760

**Published:** 2023-07-18

**Authors:** Chung-Chia Chang, Sung-Yen Lin, Chun-Kuan Lu, Jesse B. Jupiter, Yin-Chih Fu, Wen-Chih Liu

**Affiliations:** 1 Department of Orthopedic Surgery, Kaohsiung Medical University Hospital, Kaohsiung Medical University, Kaohsiung 80756, Taiwan; luckychungchia@gmail.com (C.-C.C.); tony8501031@gmail.com (S.-Y.L.); microfu@gmail.com (Y.-C.F.); 2 School of Post-Baccalaureate Medicine, College of Medicine, Kaohsiung Medical University, Kaohsiung 807378, Taiwan; 3 Orthopaedic Research Center, Kaohsiung Medical University, Kaohsiung 807377, Taiwan; 4 Regeneration Medicine and Cell Therapy Research Center, Kaohsiung Medical University, Kaohsiung 807377, Taiwan; 5 Department of Orthopedic, Park One International Hospital, Kaohsiung 813017, Taiwan; u9001054@yahoo.com.tw; 6 Hand and Arm Center, Department of Orthopedic Surgery, Massachusetts General Hospital, Harvard Medical School, Boston, MA 02114, USA; 7 Department of Orthopedic Surgery, Kaohsiung Municipal Ta-Tung Hospital, Kaohsiung 801735, Taiwan; 8 Ph.D. Program in Biomedical Engineering, College of Medicine, Kaohsiung Medical University, Kaohsiung 807378, Taiwan; 9 School of Medicine, College of Medicine, Kaohsiung Medical University, Kaohsiung 807378, Taiwan

**Keywords:** volar plate arthroplasty, volar plate interposition arthroplasty, post-traumatic osteoarthritis, hand injury, joint preservation, proximal interphalangeal joint

## Abstract

This is a retrospective study to evaluate the outcome of volar plate interposition arthroplasty for proximal interphalangeal joint post-traumatic osteoarthritis with a minimum 5-year follow-up. We identified patients receiving volar plate interposition arthroplasty for post-traumatic osteoarthritis in proximal interphalangeal joints. The measurements included the numeric pain scale (on a scale of 0–10), the proximal interphalangeal joint active range of motion, the Michigan Hand Outcomes Questionnaire, the perioperative radiograph of the involved digit, proximal interphalangeal joint stability, and pinch strength. Eight patients with a median age of 44 years old (interquartile range (IQR): 29.3–56.8) were included in this study. The median follow-up period was 6.5 years (range of 5–11 years). The median numeric pain scale improved from 5 (IQR: 4.3–6.0) preoperatively to 0 (IQR 0–0.8) at the follow-up evaluation (*p* = 0.011). All digits demonstrated stability during manual stress testing compared to their noninjured counterparts. The median active proximal interphalangeal joint arc of motion improved from 25° to 55° (*p* = 0.011). The pinch strength of the fingers on the injured hand was weaker than those on the contralateral hand (2.2 Kg vs. 3.7 Kg, *p* = 0.012). We suggested that volar plate interposition arthroplasty may be an alternative surgical option for post-traumatic osteoarthritis in the proximal interphalangeal joints.

## 1. Introduction

Arthritis is a commonly encountered pathological ailment that prominently affects the hand and its digits. It typically manifests sequentially, primarily targeting the distal interphalangeal joints, followed by the involvement of the carpometacarpal joints, metacarpophalangeal joints, and proximal interphalangeal joints [1]. Arthritis encompasses two major subtypes: inflammatory arthritis and osteoarthritis. Within the osteoarthritis category, a further subdivision is recognized, including primary and secondary osteoarthritis, also called post-traumatic osteoarthritis. Primary osteoarthritis commonly involves distal interphalangeal joints and carpometacarpal joints. The propensity for post-traumatic osteoarthritis development is higher in the proximal interphalangeal joint than in other joints, primarily attributable to its elevated fractional dissipative energy and increased cartilage cell death after an injury [2].

Post-traumatic osteoarthritis of the hand, particularly in the proximal interphalangeal and metacarpophalangeal joints, may interfere with a patient’s routine due to joint pain and restricted range of motion of the digits. At the beginning of the disease, conservative treatment options include oral analgesics, anti-inflammatory medication, and intra-articular injections [3]. For those patients who have undergone unsuccessful conservative treatment for at least six months, the operative treatment, including synovectomy and joint denervation [4,5,6,7,8], are available, alternative options that can lead to optimal outcomes. Synovectomy may be chosen in the early stage of osteoarthritis to subside the inflammation in those cartilage-preserved joints [4]. Joint denervation for post-traumatic osteoarthritis has been shown to be effective for proximal interphalangeal joints with an improved numeric pain scale and disabilities of the arm, shoulder, and hand questionnaire [5]. In a painful joint with severe functional limitations, surgical treatment options include joint fusion [9], implant arthroplasty [9,10,11,12,13], and free vascularized joint transfer [14,15]. Arthrodesis, including K-wires, tension band wiring, plates, and headless compression screws, may provide excellent pain relief and stability for those joints in the end stage of osteoarthritis [9]. Nevertheless, the complete loss of joint motion may cause some functional limitations [16]. To preserve joint mobility, implant arthroplasty plays a role in treating finger osteoarthritis; however, some complications following arthroplasty include instability, implant breakage, implant loosening, and dislocation [10,11]. The free vascularized joint transfer is a technically challenging procedure that improves the range of motion and patient-reported outcome measures [17,18]. As stated above, determining the ideal treatment to alleviate pain and simultaneously address stability and range of motion is still challenging among surgeries.

Volar plate arthroplasty was initially developed to fill the osteochondral defect of the finger joint, particularly in individuals with fracture dislocations [19]. Burton et al. published a preliminary report with encouraging results of the technique when applied to proximal interphalangeal joints with osteoarthritis [20]. In their study, the collateral ligaments were excised, so the procedure was only indicated in relatively stable joints. Lin et al. reported a modification of the surgical technique and emphasized the role of “interposition”, so-called volar plate interposition arthroplasty, and the created space provided a better range of motion in addition to the established benefits [21]. The pain relief and functional preservation outcomes with a minimum 2-year follow-up are satisfactory. However, we are uncertain if the interposed volar plate can endure longer-term stress and cause changes in functional outcomes. Therefore, this study aims to present a cohort with a minimum 5-year follow-up after receiving volar plate interposition arthroplasty for post-traumatic osteoarthritis of the proximal interphalangeal joints.

## 2. Materials and Methods

### 2.1. Patient Selection

We reviewed the electronic medical record of the Department of Orthopedic Surgery at Kaohsiung Medical University Hospital in April 2023. The Institutional Review Board of Kaohsiung Medical University Hospital (KMUHIRB-E(I)-20230043) approved this retrospective observational study. The inclusion criteria were patients who received volar plate interposition arthroplasty for post-traumatic osteoarthritis of the proximal interphalangeal joint with a minimum 5-year follow-up with radiological and functional results. The diagnosis of post-traumatic osteoarthritis was made radiographically since arthritic changes at the joint suggested posttraumatic symptoms for over six months after a trauma episode. The indications of volar plate interposition arthroplasty for post-traumatic osteoarthritis were joint space narrowing in either anteroposterior or lateral views of the proximal interphalangeal joints and patients suffering from persistent pain and limited range of motion. Those patients with severely unstable joints with poor soft tissue envelopes and incompetent collateral ligaments, such as those with rheumatoid arthritis, were not included in this cohort. A single senior surgeon performed all the procedures.

### 2.2. Surgical Technique

The arthritic joint was opened with a volar incision. The volar plate was incised along the margin and detached as distally as possible to preserve adequate length for advancement into the joint. Osteophytes at the joint were removed, whereas the bilateral collateral ligaments were preserved. To reshape the good congruent articulation of the head of the proximal phalanx and the base of the distal phalanx, we used a small curette to remove the remaining frayed articular cartilage, followed by the placement of the volar plate within the joint space. To avoid iatrogenic joint instability, we suggest meticulously resurfacing the joint with a small rongeur or curette, piece by piece, until no mechanical block is noted during the joint’s passive range of motion. The two ends of the volar plate are tagged with nonabsorbable 4–0 sutures and reflected proximally.

To anchor the volar plate on the dorsal apparatus of the proximal interphalangeal joint and smoothly spread it over the joint surface like a parachute, we passed two 19-gauge needles parallel or slightly divergent from the volar side, at the most radial and ulnar borders of the proximal interphalangeal joint, to the dorsal side. Then, the other two 19-gauge needles were placed at the tips of the previously mentioned two needles and then directed back into the proximal interphalangeal joint from the dorsum to the volar proximal interphalangeal joint. Next, the two ends of the suture that had already been stitched on the edges of the volar plate were threaded through these two needles, bringing the sutures from the volar side through the dorsal apparatus and out to the dorsal skin surface of the proximal interphalangeal joint. A small horizontal incision was made in the dorsal skin over the proximal interphalangeal joint between the locations where the two stitches emerged.

**Figure 1:**
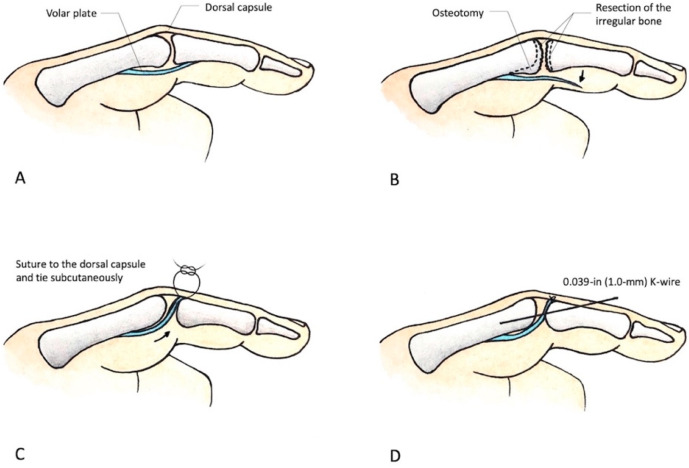
The schematic diagram of volar plate interposition arthroplasty: (**A**) The anatomy of the normal proximal interphalangeal joint. (**B**) The volar plate was detached as distally as possible. Parts of the bone in the distal condyle of the proximal phalanx for proximal interphalangeal joints were resected, and so were the irregular bones over the destructive joint’s surface. (**C**) The volar plate was interposed into the joint and sutured to the dorsal capsule with a redundant length of the suture line left at both ends. The ends of the suture were tied together, and a small transverse skin incision was made to hide the node subcutaneously. (**D**) A 0.039 inch (1.0 mm) K-wire was placed across the joint at 20° flexion to provide additional stability.

**Figure 2:**
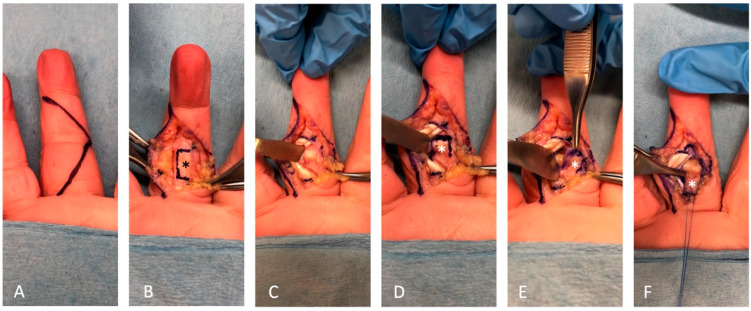
The volar plate interposition arthroplasty procedure in a cadaver specimen: (**A**) Volar approach with Bruner incision. (**B**) The flexor sheath was exposed after the elevation of the skin flap. The distal edge of the A2 pulley and the proximal edge of the A4 pulley were identified. The C1, A3, and C2 flaps (black asterisk) were designed from the edges to expose the flexor tendons without A2 and A4 injuries. (**C**) The flexor tendons were retracted to one side, and (**D**) the proximal interphalangeal joint’s volar plate (white asterisk) was accessed. (**E**) The volar plate (white asterisk) was further incised along the margin, detached as distally as possible, and (**F**) was ready to be interposed into the joint and sutured to the dorsal capsule with a redundant length of the suture left at both ends.

Sometimes, the volar plate may be insufficient in length. In such cases, we have found it necessary to secure the knot at 20° to 30° of proximal interphalangeal joint flexion to ensure optimal positioning of the volar plate within the joint space, extending towards the dorsal capsule. Afterward, we tied the knot on the extensor hood, closing the wound and allowing the knot to remain subcutaneous. Finally, a 1.0 mm K-wire was placed across the joint at 20° to 30° of flexion to provide additional stability in the proximal interphalangeal joint. Figure 1 displays the schematic diagram illustrating the technique of volar plate interposition arthroplasty, while Figure 2 showcases the surgical procedure involving retrieving the volar plate from a cadaver specimen.

The K-wire was extracted two weeks after the surgery. Subsequently, the patient was granted permission to initiate an active range of motion under the supervision of an extension-block splint, limited to 20° flexion, for an additional two-week period. Following this, the patient’s range of motion restrictions was lifted, eliminating the need for an extension-block splint, and thereby enabling complete and unrestricted joint movement.

### 2.3. Outcome Measure

The medical records and radiographs of each patient were reviewed retrospectively. All patients returned to outpatient clinics for follow-up, where a single resident surgeon (C, C.-C) performed a complete physical examination of the operative finger. We conducted a comprehensive follow-up assessment of patients at our clinic, meticulously and prospectively documenting all pertinent clinical data. The patient-reported outcomes were evaluated using the numeric pain scale of 0 to 10 and the Michigan Hand Outcomes Questionnaire score [22]. The numeric pain scale ranged from 0 (no pain) to 10 (worst possible pain). They were compared with the preoperative values routinely recorded in patients’ medical charts by the operating surgeon at the preoperative visit. The Michigan Hand Outcomes Questionnaire scores ranged from 0 (the worst) to 100 (the best) and were only evaluated at the final follow-up.

The objective assessments evaluated joint alignment per radiograph, active range of motion, joint stability (as determined by manual testing at 30° flexion), and pinch strength of the injured and noninjured fingers (pulp of the test finger to the pulp of the thumb). All examinations were performed by a single examiner who was distinct from the operating surgeon. The active motion arc over the operated joint was measured with a digital goniometer, defined as the degree of full extension subtracted from the active maximal flexion.

The coronal alignment of the joint was defined as the intersection angle between the middle and proximal phalanx axes on the anteroposterior radiograph. Joint stability was examined through manual stabilization of the proximal phalanx, followed by applying manual stress to the finger distal to the operated joint on the volar–dorsal and radial–ulnar axes. Any joint subluxation or dislocation related to manual stress was defined as instability. In testing pulp pinch strength, patients were seated with their shoulders adducted and neutrally rotated, their elbows flexed at 90°, and both the forearm and wrist in the neutral position. All measurements were performed by Jamar^®^ 50-pound pinch gauge.

### 2.4. Statistical Test

Descriptive statistics were computed for each of the variables, namely median and interquartile range (IQR), depending on the presence of nonnormal distribution (n < 20). The Wilcoxon matched-pairs signed-rank test was used to compare the values of the numeric pain scale and the active arc of motion before surgery and at the final follow-up evaluation, and the pulp pinch strength between postoperative injury and contralateral noninjured fingers at the final follow-up. A significance level of *p* = 0.05 was used for each statistical test.

## 3. Results

In this retrospective study conducted at Kaohsiung Medical University Hospital, an analysis of medical records was undertaken. A total of 30 patients who were diagnosed with post-traumatic osteoarthritis affecting the digits and underwent volar plate implant arthroplasty between 2004 and 2018 were identified. Among this cohort, only eight patients with post-traumatic osteoarthritis specifically affecting the proximal interphalangeal joint completed the minimum 5-year follow-up assessment, thereby providing valuable long-term data for analysis.

The demographic data of the eight patients included seven males and one female, with a median age of 44 years old (IQR: 29.3–56.8) at the time of receiving the procedure. The patients’ median follow-up period was 6.5 years (IQR: 5–7.8). Furthermore, the median time elapsed between the trauma episode and the receipt of volar plate interposition arthroplasty was 12 months (IQR: 7.2–21.6). Three of this cohort’s injured digits (37.5%) were in the dominant hand. Examining the initial mechanisms of injury, it was observed that three cases involved crushing injuries of the proximal interphalangeal joints; two cases were proximal phalangeal condylar fractures; two cases were middle phalanx base intraarticular fractures; and one case was a traumatic amputation (occurring 1 cm distal to the proximal interphalangeal joint) that underwent replantation surgery. Notably, all affected fingers had undergone surgical repair, replantation, and stabilization after the initial injury.

A subjective assessment of the numeric pain scale revealed noteworthy improvements. The numeric pain scale showed a median value of 5 (IQR: 4.3–6.0) preoperatively, which decreased significantly to 0 (IQR: 0–0.8) during the follow-up evaluation (*p* = 0.011). This reduction in pain following surgery was statistically significant. What was particularly remarkable was that individuals who scored zero on the numeric pain scale reported experiencing no pain whatsoever, even during rest, activity, and weather changes. Furthermore, the median score on the Michigan Hand Outcomes Questionnaire score was determined to be 76.5 (IQR: 66.5–89.5) at the final follow-up. This score provides an overall assessment of hand function and quality of life.

**Figure 3:**
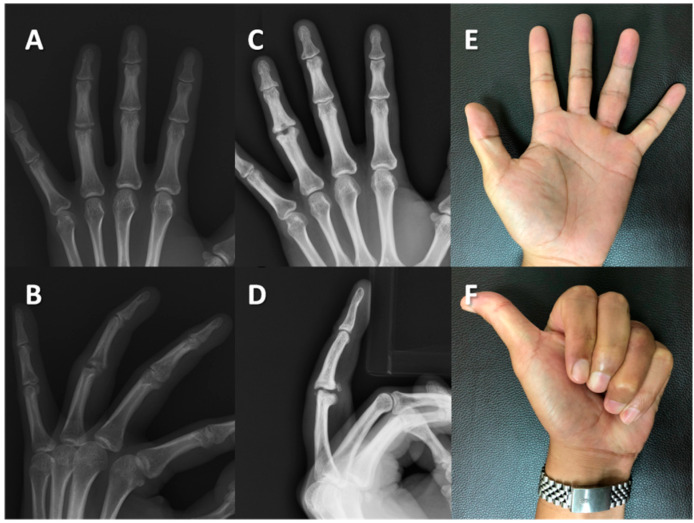
Case #5: A 33-year-old man developed post-traumatic osteoarthritis of the left ring finger. Radiographs of the left ring finger in the (**A**) anteroposterior (AP) view and (**B**) lateral view revealed post-traumatic osteoarthritis of the proximal interphalangeal joint in the preoperative status. The six-year follow-up assessment revealed preserved joint space in the (**C**) AP and (**D**) lateral views. The active flexion of the finger was 90° (**E**) with no extensor lag (**F**).

**Figure 4:**
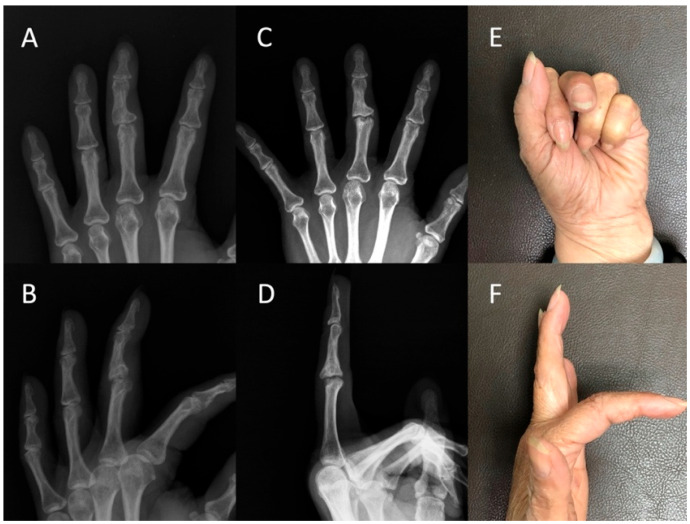
Case #8: A 57-year-old woman developed left middle finger post-traumatic osteoarthritis. Radiographs of the left middle finger in the (**A**) anteroposterior (AP) view and (**B**) lateral view revealed post-traumatic osteoarthritis of the proximal interphalangeal joint in the preoperative status. The five-year follow-up assessment demonstrated the preservation of joint space in the (**C**) AP and (**D**) lateral views. The active flexion of the finger was 40° (**E**) with no extensor lag (**F**).

The objective assessment examined the median active arc of motion of the proximal interphalangeal joints. The preoperative value was measured to be 25° (IQR: 11.3–43.8), which demonstrated significant improvement to 55° (IQR: 41.3–67.5) at the final follow-up (*p* = 0.011). Moreover, the postoperative median extensor lag of the proximal interphalangeal joints was 0° (IQR: 0–17.5), while the median active flexion measured 60° (IQR: 41.3–85.0). In terms of coronal alignment, the preoperative assessment showed a median value of 7.9° (IQR: 3.6–17.3), whereas the alignment observed in the follow-up radiograph yielded a median value of 7.4° (4.2–12.0) (*p* = 0.575). It is worth noting that all digits demonstrated stability during manual stress testing compared to their noninjured counterparts. However, the pinch strength of the postoperative fingers was weaker, measuring 2.2 Kg compared to the contralateral fingers’ 3.7 Kg (*p* = 0.012). To provide a comprehensive overview of the findings, the results of both preoperative and postoperative clinical assessments have been presented in Table 1. We presented the radiographs and clinical outcomes of two injured fingers in Figure 3 and Figure 4.

## 4. Discussion

There are various surgical treatment options available for proximal interphalangeal joint post-traumatic osteoarthritis. These options encompass [6], joint denervation [5,6,7,8], arthrodesis [9], implant arthroplasty [10,11,12], and free vascularized joint transfer [14,15]. This study consisted of a cohort with post-traumatic osteoarthritis of the proximal interphalangeal joints who underwent volar plate interposition arthroplasty and were followed up for a minimum of 5 years. The functional range of motion improved from 25° to 55°, and the numeric pain scale decreased from five to zero. At the final follow-up, the median Michigan Hand Outcomes Questionnaire score was 76.5, and the pinch strength was 2.2 kg compared to 3.7 kg in the noninjured counterpart. Our findings indicate that volar plate interposition arthroplasty provides measurable functional outcomes and pain relief benefits.

Synovectomy may be applied in the early stages of osteoarthritis [4]. According to Gschwend et al. [5], the best indication for synovectomy is patients with outstanding responses to intra-articular steroid injections and less than 20% of cartilage injury in the affected joints. However, this indication could only be checked during an operation. Moreover, the success rate of synovectomy alone could be more impressive even in this selected patient group. Joint denervation is a simple technique to relieve pain in osteoarthritis. Research showed that the outcome was satisfactory, with about 80% pain relief. However, it is recommended for joints that exhibit a preserved preoperative range of motion and demonstrate satisfactory lateral stability [7].

For those joints in the end stage of osteoarthritis, especially with pre-existing deformity and instability in the digits, arthrodesis could provide significant pain relief and stability. Although fine motor skills are affected by limited finger range of motion, arthrodesis of the digits in a functional position still provides adequate function for low-demanded activities of daily living [3]. Several arthrodesis techniques include interosseous wiring, tension-band wiring, plate fixation, and screw arthrodesis [9]. The common complications of arthrodesis include delayed union, nonunion, deep infection [23,24], and limited grip strength [25]. Complete loss of joint motion may cause some functional limitations [16], especially in patients with high finger functional demands.

In comparison to arthrodesis, implant arthroplasty may preserve the range of motion. More and more surgeons choose arthroplasty rather than arthrodesis because a proximal interphalangeal arthroplasty gives better function than a fusion, even with limited mobility [3]. Several implants are available for arthroplasty, but only a few have proven adequate with long-term follow-up. Silicone implants are still the gold standard with acceptable long-term outcomes, even with the risks of implant breakage and rare silicone synovitis [10]. Newer resurfacing-type designs, such as pyrocarbon implants, were reported to have risks of dislocation and implant loosening [11]. Surface replacement arthroplasty is another option for primary osteoarthritis of the digits; however, early contracture and osteophyte formation may cause deterioration in the range of motion of the proximal interphalangeal joint [26]. Some researchers even showed a 4.3 times increased risk of complication in patients undergoing arthroplasty versus arthrodesis [27]. Thus, the pros and cons of preserving the joint range of motion with arthroplasty should be carefully weighed.

The vascularized joint transfer has been described as a recommended finger joint reconstruction with adequate functional restoration. Loh et al. reported the results on 38 fingers followed up over an average of 27 months (range: 6–123 months), and the active arc of motion was 58° [14]. However, other studies have reported a mean active arc of motion of approximately 37° [28,29]. In addition, a vascularized joint transfer is technically demanding, and optimal outcomes are only obtained with a concurrent extensor reconstruction.

Volar plate arthroplasty was initially considered a treatment for acute fracture dislocation of proximal interphalangeal joints, in which accurate joint congruity was impossible to repair [30]. The indications were gradually expanded to include long-term persistent or neglected fracture dislocation, osteoarthritis of proximal interphalangeal joints [16], and rheumatoid arthritis of metacarpophalangeal joints [31]. In the acute stage of dorsal fracture dislocation, volar plate arthroplasty has been suggested to be beneficial on a long-term basis [32]. However, the technique has rarely been reported in post-traumatic osteoarthritis of finger joints, and more information should be available regarding the indications and outcomes. Compared to traditional volar plate arthroplasty and the volar plate interposition arthroplasty in our previous report, we modified the volar plate interposition arthroplasty by sewing the volar plate directly onto the dorsal capsule rather than covering the destructive articular surface of the middle phalanx [20] or making bony tunnels on the proximal phalanx to pass sutures and tie to the volar aspect of the bone [19].

The mechanism of stabilization maintenance of the volar plate interposition arthroplasty is based on creating a congruent joint after reshaping the irregular proximal phalanx surface to the middle phalanx base in both the coronal and sagittal planes. The volar plate, a thick layer of durable fibrocartilage, interposes in the proximal interphalangeal joint function as a spacer after reshaping and does not influence the coronal stability. We propose that the main reason is that the interposed volar plate could be sewn onto the dorsal capsule hood, which halts the tendency of the middle phalanx to glide dorsally. The dorsal capsule and extensor hood attached to the base of the middle phalanx prevent the middle phalanx from gliding volarly. As a result, we did not notice hyperextension instability caused by volar plate interposition arthroplasty in our cohort of a longer-term follow-up. Surgeons should prevent excessive resurfacing of the proximal interphalangeal joints, which might shorten the phalanx and influence the osteokinematics of the proximal interphalangeal joints. However, excessive resurfacing is sometimes unpreventable in some severely deformed cases, resulting in unsatisfactory outcomes.

The reconstruction of joint congruity was achieved in volar plate interposition arthroplasty by resurfacing the arthritic joint. Although the range of motion (55°) may be better than that achieved by an arthrodesis and was comparable to that of implant arthroplasty, it still needs to be determined whether that range is a functional benefit to patients from our results, especially in the absence of any preoperative functional outcome. The mean arc of motion of volar plate interposition arthroplasty for proximal interphalangeal joints was 76° in the previous study at a 2-year follow-up [21]; in contrast, the arc of motion of volar plate interposition arthroplasty for proximal interphalangeal joints in this cohort was 55° with a minimum 5-year follow-up. Although the patient cohort in this study differed from the cohort in the previous study [21], it is worthwhile for surgeons to know that the arc of motion of volar plate interposition arthroplasty for proximal interphalangeal joints may change over time.

Some other literature presented a similar procedure to volar plate interposition arthroplasty. Faccio et al. described a 49-year-old female with diffuse arthritis of the finger proximal interphalangeal joints using volar plate interposition arthroplasty to realize excellent stability and a range of motion at a 6-month follow-up [33]. As they described in the report, they had some details that differed from the procedure mentioned in this study. For example, they did not suture the volar plate to the dorsal capsule, did not place K-wire through proximal interphalangeal joints, did not use pull-out sutures to secure the volar plate to the middle phalanx, and only let the volar plate fill the recipient bed. They believed those modified details took advantage of immobilization and spontaneous scarring to achieve a better postoperative outcome. The short-term outcome seems satisfactory, but further long-term follow-up should be tracked to see if the stiffness-free range of motion can be preserved. The other case report from Hidajat et al. [34] presented a 12-year-old girl with post-traumatic osteoarthritis in the proximal interphalangeal joint of her left ring finger. Unlike the surgical method described in this study, they use periosteum as an interposition material. The postoperative follow-up assessment revealed a notable enhancement in the range of motion of the proximal interphalangeal joint, accompanied by significant pain alleviation during the 12-week evaluation. Given these positive short-term results, it is imperative to continue observing the long-term outcome, as it holds considerable clinical significance.

We could not claim whether this treatment is better or worse than other strategies for reconstructing this problematic injury. We suggested that the treatment of an osteoarthritic proximal interphalangeal joint may present differently. It would need to be treated individually based on factors such as the extent of soft tissue contracture, joint destruction, and the patient’s interests and requirements. We suggested that volar plate interposition arthroplasty could be applied to post-traumatic osteoarthritis in a younger population with less soft-tissue contracture and milder destructive joints.

This retrospective study possesses inherent limitations that are commonly observed in such investigations. Firstly, the absence of preoperative patient-reported outcome measures prevents us from drawing definitive conclusions regarding the additional benefits of this procedure on the overall hand function. Secondly, the long-term follow-up data were unavailable for all patients, and the enrollment of participants was constrained due to the stringent requirement of a minimum five-year follow-up period, potentially introducing a selection bias. Furthermore, being a single-center study, the number of eligible patients meeting the inclusion criteria was further reduced. Finally, due to the relative rarity of post-traumatic osteoarthritis affecting the proximal interphalangeal joint, conducting a comparative study to assess alternative surgical interventions, such as arthrodesis, implant arthroplasty, or free vascularized joint transfer, in comparison to volar plate interposition arthroplasty, poses significant challenges. Consequently, the superiority of volar plate interposition arthroplasty in terms of outcomes for post-traumatic osteoarthritis affecting the proximal interphalangeal joint remains indeterminate.

In conclusion, volar plate interposition arthroplasty emerges as a viable surgical alternative for addressing post-traumatic osteoarthritis in finger joints, effectively addressing the primary objectives of pain alleviation, stability preservation, and functional restoration. Volar plate interposition arthroplasty is a relatively simple surgical approach, delivering outcomes comparable to other surgical treatments for post-traumatic osteoarthritis in the proximal interphalangeal joints. Therefore, it can be considered a suitable alternative for PTOA when implants are unavailable, microsurgical reconstruction techniques cannot be performed, and the joint should be preserved.

## Figures and Tables

**Table 1:** Results of preoperative and long-term follow-up clinical assessments.

No.	Age	Sex	TV (y)	F/u (y)	Involved PIPJ	Dominant Hand	Pain Scale		Function: Arc (ROM) (°)		Coronal Alignment		Stability	MHQ	Pinch Strength (Kg)	
							Pre-VPIA	F/u	Pre-VPIA	F/u	Pre-VPIA	F/u	F/u	F/u	OP-Finger	Contralateral
1	28	M	0.5	11	L index	RHD	8	0	15 (30–45)	70 (20–90)	4.3	0.4	Stable	100	5.5	6.2
2	21	M	1	8	L index	RHD	6	3	0 (fixed 10)	10 (10–20)	19.4	14.6	Stable	76	4.5	6.8
3	62	M	5	7	L small	RHD	4	0	45 (0–45)	60 (0–60)	27.7	6.0	Stable	82	1.4	2.9
4	56	M	2	7	R ring	RHD	5	0	40 (20–60)	50 (20–70)	8.7	9.7	Stable	77	2.9	4.5
5	33	M	1	6	L ring	RHD	5	0	30 (0–30)	90 (0–90)	7.0	4.5	Stable	92	3.2	4.6
6	47	M	0.5	5	L ring	RHD	6	0	20 (0–20)	45 (0–45)	10.9	12.8	Stable	53	0.5	2.3
7	41	M	1	5	R small	RHD	5	1	50 (0–50)	60 (0–60)	1.1	4.0	Stable	64	1.4	1.8
8	57	F	1	5	L middle	LHD	4	0	10 (0–10)	40 (0–40)	3.3	8.8	Stable	74	0.5	1.4
Median	44		1	6.5			5	0	25	55	7.9	7.4		76.5	2.2	3.7
IQR	29.3, 56.8		0.6, 1.8	5, 7.8			4.3, 6.0	0, 0.8	11.3, 43.8	41.3 67.5	3.6, 17.3	4.2, 12.0		66.5, 89.5	0.7, 4.2	1.9, 5.8
*p*-value *							0.011		0.011		0.575				0.012	

TV, trauma-to-VPIA period; F/u, follow-up; VPIA, volar plate interposition arthroplasty; ROM, active range of motion; MHQ, Michigan Hand Outcomes Questionnaire; M, male; F, female; RHD, right hand dominant; LHD, left hand dominant; L, left; R, right; PIPJ, proximal interphalangeal joint; IQR, interquartile range (25th and 75th percentile). * *p*-value, determined using Wilcoxon matched-pairs signed-rank test.

## Data Availability

The data presented in this study are available in articles.
